# Local Allergic Rhinitis—A Challenge for Allergology and Otorhinolaryngology Cooperation (Scoping Review)

**DOI:** 10.3390/life14080965

**Published:** 2024-07-31

**Authors:** Ovidiu Berghi, Mihai Dumitru, Romica Cergan, Gabriela Musat, Crenguta Serboiu, Daniela Vrinceanu

**Affiliations:** 1 Allergology Department, Colentina Clinical Hospital, 020125 Bucharest, Romania; oberghi@yahoo.com; 2 ENT Department, Faculty of Medicine, Carol Davila University of Medicine and Pharmacy, 020021 Bucharest, Romania; vrinceanudana@yahoo.com; 3 Anatomy Department, Faculty of Medicine, Carol Davila University of Medicine and Pharmacy, 020021 Bucharest, Romania; r.cergan@gmail.com; 4 ENT Department, Faculty of Dentistry, Carol Davila University of Medicine and Pharmacy, 020021 Bucharest, Romania; gabimusat@yahoo.com; 5 Histology and Molecular Biology Department, Carol Davila University of Medicine and Pharmacy, 020021 Bucharest, Romania; crengutas@yahoo.com

**Keywords:** local allergic rhinitis, allergology, ENT

## Abstract

Local allergic rhinitis (LAR) represents a medical provocation for allergists and otorhinolaryngologists. LAR is considered to be a subtype of allergic rhinitis (AR) that affects a great percentage of patients who were, for decades, diagnosed as having chronic non-allergic rhinitis. The clinical picture is represented by rhinorrhea, sneezing, and nasal itching correlated with specific pollen season or dust, mold, or pet interior exposure. Usual assessment of AR (skin prick testing and serum IgE assessment) produces negative results. Specialized centers in allergology and ENT around the globe use a nasal allergen challenge, assessment of local IgE, basophil activation test (BAT), and nasal cytology in the diagnostic approach to the disease, taking into account their current limitations. The impact of LAR on quality-of-life indicators is the same as in AR. Treatment for LAR is similar to that for AR and is the same as for AR: allergen exposure avoidance, drug therapy, and allergen immunotherapy. This scoping review gathers the current up-to-date open access evidence available on PubMed on the subject of LAR.

## 1. Introduction

The first steps in defining LAR were made in the 1970s. Tse et al. detected ragweed pollen-specific IgE in nasal secretions of ragweed-sensitized patients [1]. Huggins and colleagues studied in 1975 a group of patients with classic symptoms of AR to house dust mites (HDM). All the investigations were normal: negative skin prick tests and serum IgE. They performed a nasal allergen challenge (NAC) and measured local specific IgE against HDM, proving that these patients could produce only local IgE [2]. Platts-Mills showed that allergen-specific IgG, IgA, and IgE antibodies were present in the serum and in the nasal mucosa in the majority of the grass pollen-allergic patients with higher levels in the nasal mucosa [1,3]. Later, Powe et al. proposed the term “entropy” to define the notion of localized allergic reaction detected by the presence of specific IgE antibodies in nasal secretions in non-atopic idiopathic rhinitis subjects and allergic subjects [1,4]. The notion of LAR was proposed by Professor Carmen Róndon in 2009 [5]. The objective of the present scoping review is to gather the existing state-of-the-art open access data on the subject of LAR.

## 2. Material and Method

**Figure 1:**
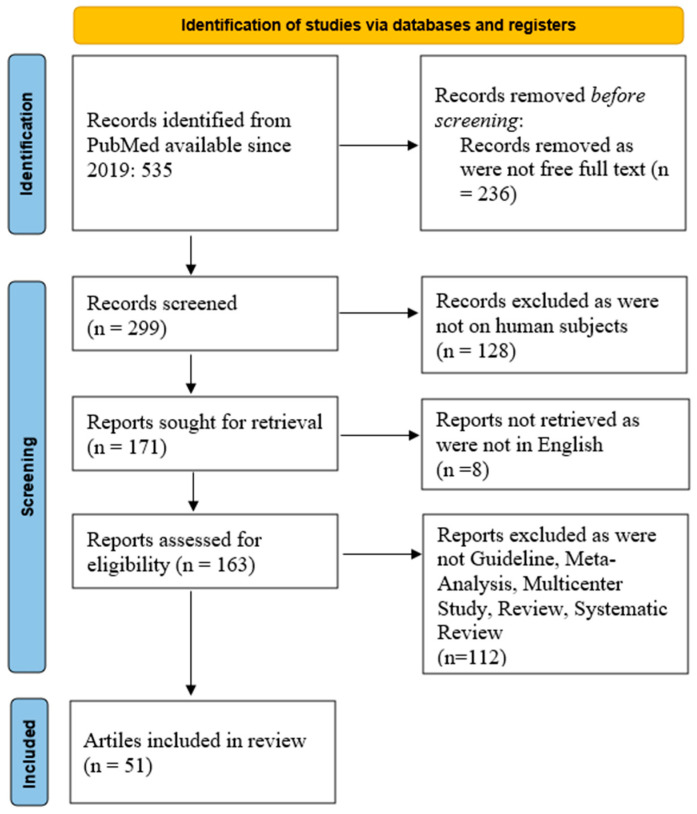
PRISMA flow chart of the present scoping review on LAR.

We queried the PubMed database with the key words: local allergic rhinitis. We retrieved 535 articles since 2019. From these records, only 299 articles were available for free as full text. Further restricting the search to manuscripts on human subjects, we retrieved 171 articles. From these articles, only 163 were available in the English language. Further, we restricted the search to Guideline, Meta-Analysis, Multicenter Study, Review, Systematic Review, and we included 51 articles in the current search, Figure 1. The search syntax was: ((“focal”[All Fields] OR “focalities”[All Fields] OR “focality”[All Fields] OR “focalization”[All Fields] OR “focalized”[All Fields] OR “focally”[All Fields] OR “focals”[All Fields] OR “local”[All Fields] OR “localisation”[All Fields] OR “localisations”[All Fields] OR “localise”[All Fields] OR “localised”[All Fields] OR “localises”[All Fields] OR “localising”[All Fields] OR “localization”[All Fields] OR “localizations”[All Fields] OR “localize”[All Fields] OR “localized”[All Fields] OR “localizer”[All Fields] OR “localizers”[All Fields] OR “localizes”[All Fields] OR “localizing”[All Fields] OR “locally”[All Fields] OR “locals”[All Fields]) AND (“rhinitis, allergic”[MeSH Terms] OR (“rhinitis”[All Fields] AND “allergic”[All Fields]) OR “allergic rhinitis”[All Fields] OR (“allergic”[All Fields] AND “rhinitis”[All Fields]))) AND ((y_5[Filter]) AND (ffrft[Filter]) AND (guideline[Filter] OR meta-analysis[Filter] OR multicenterstudy[Filter] OR review[Filter] OR systematicreview[Filter]) AND (humans[Filter]) AND (english[Filter])).

We hope to grow the awareness of ENT and allergology specialists in the expanding field of local allergic rhinitis. The present scoping review could be a cornerstone for developing further interdisciplinary teams for research on specific allergens.

We followed the steps outlined by Tricco et al. in designing this scoping review: (1) Identify the research question, (2) identify relevant studies, (3) select studies using an iterative team approach, (4) chart the data incorporating numerical summary and qualitative thematic analysis, (5) summarize and report the results, and (6) perform a consultation exercise, which is an optional step, but it helps in presenting the implications for policy, practice, or research [6].

PubMed search results outlined in Figure 1 were imported into one online cloud database. Subsequently, two groups of two reviewers (O.B., M.D., and C.S., D.V.) screened titles, abstracts, and full texts for inclusion, independently. All discrepancies between reviewers were resolved by another set of two reviewers (R.C. and G.M). This type of reviewer distribution and control is one of the solutions to limit possible bias [7].

Our scoping review may be subject to an evidence selection bias because we focused on data available as free full text open access [8]. This can arise from publication bias, where data from statistically significant studies are more likely to be published free full text open access than those that are not statistically significant. The current scoping review is also susceptible to bias that arises in any of the included primary studies, although we tried to analyze each of them as thoroughly as possible. Finally, competing interests can lead to bias in favor of a particular intervention, in the present situation, we would like to expand our activity in the field of LAR, and in order to limit this, we included in the team developing the present scoping review a colleague from a different hospital (G.M.).

## 3. Epidemiology

**Figure 2:**
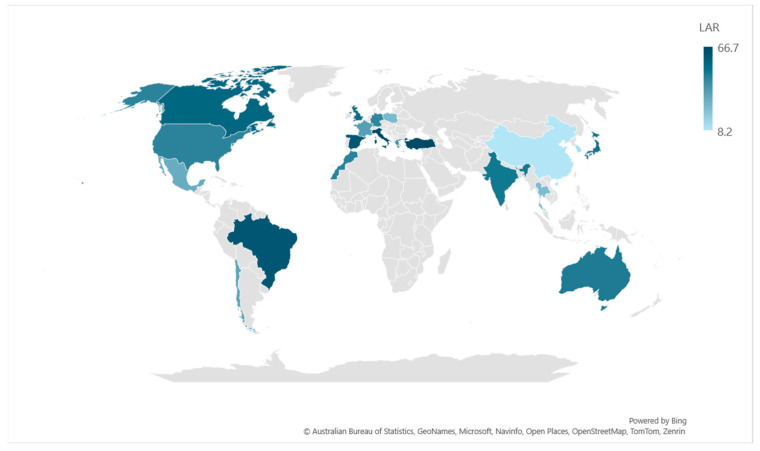
LAR prevalences in different countries. Colored countries are those for which data are available according to the studies included in this scoping review. The darkest countries are those for which the prevalence is the highest.

Variable frequencies of LAR were reported in children and adults with a lower prevalence in Asian countries [9] and higher in Mediterranean countries (Portugal, Spain, Italy, and Greece) [10]. The incidence of LAR was shown to be present in 50–75% of the population with rhinitis symptoms, without confirmed systemic atopy in Spanish studies [11,12,13]. Krajewska-Wojtys et al. diagnosed LAR in 25% of a group of 84 adults patients with chronic rhinitis and negative results of skin prick tests and sIgE tests in 2017, with house dust dominance [11,14] and Bożek found LAR in 109 (17.6%) from 621 patients with chronic rhinitis in the group with allergies mainly to house dust mites and grass pollen [11,15]. Similarly to AR, house dust mites (Dermatophagoides pteronyssinus, Dermatophagoides farinae, and other HDM) seem to be the prevalent trigger in LAR determining perennial symptoms, and grass pollen and Alternaria alternata (and less frequently other seasonal allergens such as olive tree pollen) were linked instead to seasonal LAR [2,13]. The coexistence of AR and LAR is another possible phenotype and was described by Eguiluz-Gracia et al., who proposed the term dual allergic rhinitis to define it. They pointed out that most patients with perennial rhinitis symptoms, but positive SPT for seasonal allergens only, display NAC positivity for both seasonal and perennial allergens [2,16]. These data are summarized in Figure 2.

## 4. Pathogenesis

The pathophysiology of LAR is yet to be incompletely elucidated. Immunopathology is very similar in these two entities, AR and LAR, with the main difference regarding the local nature of the allergic reaction [11]. Type 2 inflammatory response was demonstrated in the nasal mucosa of LAR patients with immediate activation of mast cells and eosinophils being observed [1]. Local IgE production against aeroallergens in nasal secretions was demonstrated both in healthy subjects and patients with AR and LAR based on nasal mucosa biopsies [2]. Allergen-specific IgE antibodies were observed in the nasal mucosa 24 h after the nasal provocation test [10]. Locally produced IgEs, after occupying all FcεRI receptors on resident effector cells, enter the systemic circulation where binding to basophil FcεRI receptors occurs. Free IgEs eventually extravasate into tissues, binding to receptors on peripheral effector cells. As a result, free IgEs are found in biological fluids only when all FcεRI receptors are saturated. In LAR patients IgE production seems to be insufficient to reach tissues outside the nose. This situation can be an explanation of why SPTs are negative, and free IgEs are undetectable in serum or absent or low in nasal secretions, whereas NAC, and usually also the basophil activation test (BAT), is positive [2]. The mast cells and eosinophils from LAR patients were found to be immediately activated in the nasal mucosa, releasing inflammatory mediators such as tryptase and eosinophil cationic protein [10]. Another emerging challenge for clinicians is taking care of rhinitis subjects overlapping LAR with NARES (nonallergic rhinitis with eosinophilia syndrome), an entity described more than 4 decades ago. Assessment of nasal eosinophils (nEo) is a preliminary marker to discriminate NARES from LAR [1,17].

## 5. Diagnostic

Diagnostic algorithms were proposed for LAR in recent years. When seeing a patient with chronic rhinitis, several steps must be conducted. The first step is taking a detailed medical history suggestive of allergic rhinitis. Skin prick tests and/or specific IgE are performed. If they are positive, a diagnostic of AR can be conducted. Nasal endoscopy and nasal/sinus computed tomography may be ordered by an otorhinolaryngologist if they are necessary. If they are negative or not correlated with the clinical picture, a diagnostic of LAR should be taken into account. A basophil activation test may be a solution if the laboratories from the surrounding area may perform it [9,10,11]. Testing for sIgE in nasal lavage fluid may be another possibility for diagnosing LAR in specialized ENT and allergology medical institutions [11]. The nasal allergen challenge is the golden standard for diagnosing LAR. The purpose of NAC is to recreate an allergic reaction in the nose after exposure to one or more aeroallergens in standardized and controlled conditions. The symptoms can be evaluated subjectively, using symptom scores and grading scales, as well as objectively, using adequate investigations [2]. Standardized methodology for the NAC was published by the European Academy of Allergy and Clinical Immunology (EAACI) in 2018 [18] and the American Academy of Allergy Asthma and Immunology (AAAAI) in 2023 [19]. The conditions in which NAC is made should be standardized, reproducible, and controlled. A nasal endoscopy should be performed before the provocation to ensure sufficient permeability of the nostrils and to rule out other inflammatory conditions and major anatomical abnormalities [20]. The examination should be carried out in a different day from the day in which the NAC is performed. Twenty minutes must be used for accommodation with the room’s atmosphere [19]. A baseline measurement of subjective (symptoms score such as Lebel or Linder scores, Visual Analogue Scale) and objective (acoustic rhinometry, peak nasal inspiratory flow, and active anterior rhinomanometry) parameters should be checked in the patient to measure if he/she are asymptomatic or suffers only from mild disease [18,20]. A control challenge (the diluent of the allergen extract) should be the next step, and nasal hyperreactivity must be excluded [20,21]. Allergen administration can be applied intranasal bilateral with a micropipette or using a nasal spray, the two most widely utilized techniques [20]. The next control must be realized 10–15 min after allergen administration. NAC should be considered positive, according to the EAACI, if moderate changes occur simultaneously in objective and subjective parameters or if clear changes are seen in at least one parameter. A recent article showed that a decrease of ≥25% in acoustic rhinometry (the area corresponding to the head of the lower turbinate in adults) had an excellent accuracy for the identification of allergen-specific nasal reactivity [22]. A single allergen dose is usually enough for diagnostic purposes, but in experienced centers, four different allergens per session can be evaluated using a NAC-validated protocol. The patient can be safely discharged 60 min after the positive response [18,20]. Nasal secretion analysis, nasal scraping/brushing, nasal nitric oxide (NO) measurement, and nasal biopsies may be realized during NAC. Nonglycerinated extracts are used in Europe and in the United States. Allergen extracts are purchased from companies that produce them for skin testing or immunotherapy. In Europe, some companies commercialize lyophilized standardized extracts for nasal challenge: Dermatophagoides pteronyssinus, Phleum pratense, Olea europea, Parietaria Judaica pollen, Alternaria alternata, and cat and dog dander [23]. Medications including topical and systemic antihistamines, topical and systemic corticosteroids, topical mast cell stabilizers, and tryciclic antidepressants should be avoided 4–21 days before [19]. Moreover, accessibility is a problem because NAC require a hospital setting ready to manage acute allergic reactions with possible life-threatening outcomes [24]. Regarding the discomfort during the NAC, the patients should be informed about the possibility of developing symptoms similar to their worst allergic episode [25].

## 6. Adult Studies

LAR was studied more intensely in recent decades. Powe et al. found grass pollen sensitization in non-atopic rhinitis patients in 2003 [3]. Carmen Rondon and her team realized the first complex studies about LAR. Specific IgE to Dermatophagoides farinae was demonstrated in patients with persistent nonallergic rhinitis [26] and specific IgE to grass and olive pollen was discovered in patients with idiophatic rhinitis [27]. Immediate and dual responses to a nasal allergen provocation test with Dermatophagoides pteronyssinus in LAR patients with mast cell/eosinophil activation and local presence of sIgE were demonstrated by the same team [28]. The next year, it the benefit of specific immunotherapy in LAR patients was demonstrated [29]. The prevalence of LAR studied for one year in a group of 452 adult rhinitis patients randomly selected from a total of 3860 who attended Allergy Service, Infanta Leonor Hospital, Malaga, Spain, was 25.7%, with Dermatophagoides pteronyssinus being the main sensitizing aeroallergen (60%) [30]. Chang et al. showed that nonspecific hyper-reactivity and/or localized allergy may play a role in patients with rhinitis whose SPT and NPT results are not in agreement [31]. Rondon and colleagues started a ten-year survey of patients with LAR. Monosensitization was the main allergen, with Dp and Alternaria Alternaria being the most frequent. At 5 years, monosensitization was still the main allergen with polysensitization increasing [32]. LAR was an important pathology in elderly patients [33]. A positive nasal provocation test was conducted in patients with rhinitis in Saudi Arabia in 18.7% of cases [34]. The analysis of the IgE receptor gene polymorphism in Egyptian patients found LAR in an important proportion of patients [35]. Blanca–Lopez and colleagues proved the relevance of grass pollen to LAR [36]. Subcutaneous immunotherapy was efficient in treating LAR with *Phleum pratensae*, with significant improvements in all primary and secondary clinical outcomes evaluated in that study and RQLQ score [37]. The results of a 10-year study follow-up of patients with LAR by C. Rondon and her team prove the consistency of this phenotype of this type of rhinitis [38]. A study from southern Poland found approximately 20% of patients with perennial nasal symptoms to have LAR, especially to dust mites [39]. The prevalence of LAR to HDM was low in a Korean rhinitis population with a longer duration of disease, older patients, and higher medication scores compared to other types of chronic rhinitis [40]. LAR was documented in 7.7% of patients with rhinitis from southern China with pollen monosensitization being the most frequent discovery [41]. Japanese cedar pollen and dust mites were the main allergens in a small study of LAR patients in Japan using NPT and total IgE and antigen-specific IgE (sIgE) from inferior turbinate mucosa [42]. Typical patients with LAR were older men with more perennial symptoms, monosensitized with D. pteronyssinus with the most sensitizing allergen, followed by phelum pratense pollen in a study from southern Poland [43]. The prevalence of LAR to dust mites in a study investigating Thai adults with chronic rhinitis was 24.2%, most of them having moderate-severe severity of the disease with sneezing being the significantly dominant symptom [44]. A percent of 6.3% of patients with symptoms of rhinitis investigated at the Rhinologic Clinic at Korea University Guro Hospital were diagnosed with LAR to dust mites [45] (Table 1).

Treatment for LAR is similar for allergic rhinitis. Measures of reducing allergen exposure are the first step. Treatment with topical corticosteroids and topical or oral antihistamines offers amelioration or resolution of symptoms in most patients [46]. Subcutaneous and sublingual immunotherapy for grass pollen and dust mites were safe, induced an increase in tolerance to the aeroallergen, and reduction in symptoms and rescue medication [47]. The EUFOREA Allergic Rhinitis guidelines are also available for LAR patients and supported the short-term effectiveness and safety of AIT for treating LAR [48]. A recent meta-analysis revealed that the short-term benefits of AIT included the improvements of combined symptom and medication score, quality of life, and sIgG4. However, there is a lack of studies of immunotherapy in LAR with measurement of long-term outcomes and in pediatric cases [49]. Many of the studies focus on grass pollen as a trigger to LAR. There is a need to expand the research on other triggers of LAR with the NAC test on a wider population. Even from a geographical point of view, there are some countries such as Romania in which NAC is used for LAR management in only one tertiary hospital, but data gathering and statistical analysis are currently under development. Knowledge about LAR is continuously increasing, with a detailed definition of physiopathological mechanisms and new phenotypes. More awareness of the disease should be promoted among different specialists, and future studies should be developed in multidisciplinary teams joining ENT and allergology specialists [50].

## 7. Pediatric Studies

Studies were also conducted in pediatric populations. Dust mites (*Dermatophagoides farinae* and *Dermatophagoides pteronyssinus*) were found to be the cause of LAR in 25% of pediatric patients with symptoms of rhinitis [51]. Local allergy to various pollens was discovered in a group of Polish teenagers [52]. The nasal allergen provocation test (NAPT) with dust mite and grass pollen and nasal sIgE levels were useful to diagnose LAR in children characterized with the same symptoms of allergic rhinitis but with the absence of markers of systemic atopy in Italy (12 patients from 18) [53]. A high incidence of LAR in pediatric patients previously classified as NAR was noticed in another Italian study implicating young children with rhinitis. Data show a higher value of nasal lavage fluid IgE (average of 6.005 UI/mL; range: 4.47–7.74 UI/mL) in 16 out of 26 patients of the study group. The authors observed a statistically significant difference (*p* < 0.0001) between the NAR/HC group and the LAR group, identifying a cut-off of 3.85 UI/mL [54]. A team from the joint allergy–ENT outpatient clinic of a tertiary pediatric hospital in Athens conducted a study on children seen within one calendar year (October 2016–September 2017). When the children were investigated using skin prick tests and/or sIgE, the results were AR discovered in 72.1% of the cases and NAR in 27.9% of them. After receiving multiple nasal provocation tests (M-NPT), the percentages were as follows: AR (72.1%), LAR (8.1%), and NAR (19.8%). The study demonstrated that approximately one in three children who would be given the diagnosis of NAR were proven to be suffering from LAR [55]. Three hundred sixty-one patients aged 5–17 with chronic rhinitis were included from eight centers in Poland in a cross-sectional observational study undertaken to determine the prevalence and clinical characteristics of LAR in Polish children. LAR was confirmed in 21% of patients with allergy to HDM prevailing in the LAR group (68%), grass pollen, and HDM in the DUAL group (32% and 64%). Girls were prevalent in the LAR group [56]. Matsumoto and colleagues made the first investigation of LAR in child and adolescent subjects in Latin America. NAC with more than one allergen was proven safe and viable in children having a critical role in the diagnosis of LAR. Among a subgroup of patients lacking systemic sensitization submitted to NAC, 40% (10/25) demonstrated a positive NAC with Dermatophagoides pteronyssinus and Blomia tropicalis, warranting their reclassification to LAR [57]. Matsumoto also wrote an excellent review about LAR in children. Ten articles were included in their article. Diagnosis rates of LAR were between 3.7 and 83.3% in children and adolescents previously categorized as having NAR, with lower rates in Eastern countries (3.7–16.6%) compared to Western countries (22.3–83.3%) [58] (Table 2).

## 8. Limitations and Future Development

One possible limitation of the current manuscript is that there are some articles which are not available free full text and thus were not included in the present analysis. In recent years, the pressure of “publishing not perish” shifted towards publishing the open access data so they are available to all the researchers worldwide. We consider that almost all major research on the subject of LAR published in the last 5 years was mainly published under an open access license. However, we encourage the scientific community to signal to us articles that were not available to present a scoping review through letter to the editorial board. Furthermore, making these scoping reviews available as open access free full texts will increase the visibility of the teams undertaking the complex field of LAR basic and clinical research.

There are some specific situations in which the protocol for LAR could be a viable solution; for example, the cases with rare allergic reactions, such as Salicaceae sensitization, that respond to treatment but fail to present clear diagnosis tests of allergic reaction. In these cases, the mucosal reaction is not always followed by a systemic response [59].

Another situation is the presence of local fungus allergic reactions. In the aftermath of the COVID-19 pandemics, there is an increase in the incidence of cases with rhinosinusal aspergilloma. These cases could present a long history of local fungal allergy that could be aggravated by the immune aggression of the virus. Further research of these cases from the point of view of LAR is necessary [60].

Moreover, LAR could be also an answer to the geographical variability of the allergic response. In current society, with large populations migrating from different continents in search of a better life, there are situations where these individuals are exposed to various allergens in their new country of residence. Populations from the middle east were forced to move to Europe to escape from war; this puts supplementary pressure on healthcare systems, as their exposure to different pollens in their native countries was minimum [61].

Occupational exposure is another aspect that needs further development regarding LAR. Actually, mucosal changes could be just a step in the pathophysiological chain of reactions that affect workers exposed to various allergens. The classical allergic reactions benefit from clear protocols, but individual exposures in specific industries could remain occult in spite of clinical manifestations and an altered quality of life of the worker [62].

## 9. Conclusions

LAR is an interesting and intriguing type of rhinitis. Due to its novelty, the diagnosis and treatment are challenging. Only few experts around the globe (Spain, Poland, Italy, Greece, Korea, and Brazil) are familiar with this disease. The current scoping review gathers up-to-date data, available open access on the subject of LAR and could be a future cornerstone for developing specific research on various potential new allergens. Specializing more allergists and otolaryngologists in centers with expertise in LAR and investing in proper technology might help improve the diagnosis and treatment of this type of rhinitis.

## Figures and Tables

**Table 1:** Summary of studies about LAR on adult patients.

Year	Author	Country	Patients	Results	Ref.
2003	D.G. Powe	United Kingdom	10 patients with non-atopic rhinitis 11 patients with AR 12 control patients	Grass pollen allergen in 3/10 non-atopic IR subjects. Specific antibodies in 8/11 mucosal samples from the allergic group.	[4]
2007	C. Rondon	Spain	50 patients with persistent nonallergic rhinitis 30 with persistent allergic rhinitis 30 healthy controls	Six patients (12%) with selective nasal sIgE-DP (Dermatophagoides pteronyssinus) in PNAR group; 25/30 patients presented sIgE-DP (83%) either in blood and/or nasal lavage in the PAR group.	[26]
2008	C. Rondon	Spain	32 patients seasonal IR (idiopathic rhinitis) 35 patients persistent allergic rhinitis to pollen (PAR-P) 30 patients persistent allergic rhinitis to house dust mites (PAR-HDM) 50 healthy nonatopic subjects	IR group selective nasal sIgE in 7/32 cases (21.8%), all seven to grass pollen and two to O. Europea nasal provocation test. IR group 20 subjects positive NAPT to grass (62.5%) and five to O. Europea (15.62%).	[27]
2010	S. Lopez	Spain	40 subjects with LAR to DP 50 healthy controls	A total of 60% of LAR patients—immediate response. A total of 40% of LAR patients—dual response. NAPT-DP was negative in all healthy controls.	[28]
2011	C. Rondon	Spain	20 adult patients with LAR sensitized to grass pollen 10 patients treated with preseasonal grass SCIT for 6 months and rescue medication in the spring (the SCIT group) 10 rescue medications (the control group)	SCIT improved objective and subjective parameters, immunologic data in patients with LAR to grass pollen.	[29]
2012	C. Rondon	Spain	452 adult rhinitis patients initially included 428 patients completed the study	LAR diagnosed in 25.7%, AR in 63.1%, and NAR in 11.2%.	[30]
2013	G.U. Chang	Republic of Korea	60 patients with AR 62 patients with NAR	Significant correlation between the result of SPT and the decrease in MCA and TNV after DP (Dermatophagoides pteronyssinus) challenge. Significant correlation between the result of NPT and the decrease in MCA/TNV after NS (Nasal saline) challenge.	[31]
2014	C. Rondon	Spain	Prospective 10-year follow-up baseline and after 5 years 194 patients with LAR of recent onset 130 healthy controls	Initial evaluation 261 aeroallergen sensitizations (Most common specific nasal sensitizing aeroallergen DP (51.1%), A alternate (35.2%). After 5 years 5.7% of the patients with LAR developed new nasal aeroallergen sensitization. Monosensitization was the predominant aeroallergen pattern; polysensitization increased, from 36.4% to 40.9%.	[32]
2015	A. Bozek	Poland	219 patients with a mean (SD) age of 65.81	A total of 46 (21.0%) had LAR, 88 (40.2%) had AR, and 85 (38.8%) non-AR. Dpt main sensitizing aeroallergen in patients with LAR and with AR.	[33]
2016	H. Badran	Saudi Arabia	1230 patients with rhinitis	77.8% of patients with positive SPT. 3.4% of patients with negative SPT with weak NPT. 18.7% of patients with negative SPT with strong NPT—LAR.	[34]
2016	N. Elbadawy	Egypt	129 patients with chronic rhinitis analyze the association of IgE receptor (FcεR1β) gene polymorphism with LAR	LAR constituted 24.8% of total rhinitis cases and 44.4% of non-allergic cases. Cockroach was the main sensitizing agent in LAR.	[35]
2016	N. Blanca-Lopez	2 hospitals in central Spain (Madrid and Ciudad Real)	61 patients with seasonal rhinitis and negative SPT results and undetectable serum sIgE	LAR was detected in 37 patients (61%).	[36]
2017	C. Rondon	Spain	56 patients with moderate-severe LAR to grass pollen	Phl-SCIT with a depigmented polymerized pollen vaccine or placebo for the first year and Phl-SCIT for the second one; 83% of patients treated with more than 6 months of SCIT tolerated a higher concentration of P. pratense than baseline, and 56% gave a negative NAPT.	[37]
2017	C. Rondon	Spain	the second phase of a 10-year follow-up study of a cohort of 176 patients with recent onset of LAR and 115 age- and sex-matched healthy controls prospectively evaluated from 2005 to 2016	LAR is a well-defined and independent rhinitis phenotype with a low rate of long-term incidence of AR with systemic atopy in adolescent and adult patients.	[38]
2017	A. Krajewska-Wojtys	Poland	84 patients with perennial nasal allergy symptoms	LAR was confirmed in 21 (25%) study patients; D. pteronyssinus in 19 (22.6%) patients, Alternaria in 3 (3.6%) patients, and the cat allergen in 1 (1.2%) patient.	[14]
2017	C.-G. Jung	Republic of Korea	304 rhinitis patients enrolled from November 2014 to March 2016; 74 patients with nasal hyper-reactivity and 80 patients with subclinical allergy were excluded	AR diagnosed in 69 (46.0%) patients, NAR in 75 (50.0%) patients, and LAR to HDM in 6 (4.0%) patients.	[40]
2018	X. Y. Tao	Southern China	194 patients with rhinitis 13 healthy subjects	A total of 115 were classified as allergic rhinitis (AR; 59.3%), 15 as LAR (7.7%), monosensitization; pollen was the most common sensitizing allergen 64 as non-AR (33.0%).	[41]
2019	M. Ishida	Japan	50 rhinosinusitis patients for surgery	JCP LAR diagnosed in 2 of 14 patients (14.3%), and HDM LAR in 5 of 21 (23.8%).	[42]
2019	A. Bozek	Poland	621 patients examined	LAR was diagnosed in 109 (17.6%) patients, AR was diagnosed in 251 (40.4%) Patients, and NAR was diagnosed in 261 (42%) patients.	[15]
2021	P. Tantilipikorn	Thailand	62 CR patients	NAPT-Dp was positive in 15/62 (24.2%) of female CR patients (73.3%), and the mean age of all patients was 36.1 ± 10.4 years.	[44]
2023	S.J. Kim	Republic of Korea	336 adult patients with rhinitis symptoms from October 2019 to April 2021	AR-HDM in 138 (41.1%) patients, AR to other allergens in 36 (10.7%) patients, NAR in 21 (42.0%) patients, and LAR-HDM in 21 (6.3%) patients.	[45]

**Table 2:** Summary of studies about LAR on pediatric patients.

Year	Author	Country	Patients	Results	Ref
2016	H. Duman	Turkey	28 patients 30 healthy children	NPT was positive in 7 (25%) patients.	[51]
2016	A. Krajewska-Wojtys	Poland	121 patients, ages 12–18 years old, confirmed nonallergic rhinitis, typical seasonal nasal symptoms	LAR to grass pollen (P. partense) 17(16.6%), LAR to *Artemisia* pollen 6(5.9%), and LAR to birch pollen 9 (8.9%).	[52]
2016	AM. Zicari	Italy	18 patients	A total of 12 patients (66.7%) with positive results in at least one NAPT; nasal sIgE levels for D. pteronyssinus, *p* < 0.005; D.farinae < 0.05; and L.perenne *p* < 0.05).	[53]
2017	L. Colavita	Italy	54 patients group (26 children with rhinitis symptoms and without evidence of systemic atopy) allergic rhinitis (AR) group (15 children) 13 healthy controls (HC).	IgE concentration in nasal lavage fluid may be considered a marker of LAR with a cut-off of 3.85 UI/mL for nasal lavage performed with 2 mL /nostril of physiologic saline solution (0.9% NaCl).	[54]
2019	O. Tsilochristou	Greece	86 patients	AR 72.1% LAR 8.1% NAR 19.8%	[55]
2023	A. Bożek	Poland (8 centers)	361 patients	LAR 21% of patients Systemic allergic rhinitis (SAR) 43.9% Dual allergic rhinitis (DUAL) 9.4% NAR in 33.9% of patients.	[56]
2024	F.Y. Matsumoto	Brazil	25 patients	NAR patients 15, and LAR patients 10.	[57]

## Data Availability

All data are available upon request from the corresponding author.

## References

[1] Mortada M.M., Kurowski M. (2023). Challenges in Local Allergic Rhinitis Diagnosis, Management, and Research: Current Concepts and Future Perspectives. Medicina.

[2] Melone G., Giorgis V., Di Pino M., Pelaia C., Nappi E., Heffler E., Landi M., Gelardi M., Paoletti G. (2023). Local Allergic Rhinitis: Lights and Shadows of a Mysterious Entity. Int. Arch. Allergy Immunol..

[3] Platts-Mills T.A. (1979). Local production of IgG, IgA, and IgE antibodies in grass pollen hay fever. J. Immunol..

[4] Powe D.G., Jagger C., Kleinjan A., Carney A.S., Jenkins D., Jones N.S. (2003). ‘Entropy’: Localized mucosal allergic disease in the absence of systemic responses for atopy. Clin. Exp. Allergy.

[5] Rondón C., Canto G., Blanca M. (2010). Local allergic rhinitis: A new entity, characterization and further studies. Curr. Opin. Allergy Clin. Immunol..

[6] Tricco A.C., Lillie E., Zarin W., O’brien K., Colquhoun H., Kastner M., Levac D., Ng C., Sharpe J.P., Wilson K. (2016). A scoping review on the conduct and reporting of scoping reviews. BMC Med. Res. Methodol..

[7] Jackson J.L., Kuriyama A. (2018). From the Editors’ Desk: Bias in Systematic Reviews—Let the Reader Beware. J. Gen. Intern. Med..

[8] Drucker A.M., Fleming P., Chan A.W. (2016). Research Techniques Made Simple: Assessing Risk of Bias in Systematic Reviews. J. Invest. Dermatol..

[9] Eguiluz-Gracia I., Testera-Montes A., Rondon C. (2021). Medical algorithm: Diagnosis and treatment of local allergic rhinitis. Allergy.

[10] Terada T., Kawata R. (2022). Diagnosis and Treatment of Local Allergic Rhinitis. Pathogens.

[11] Krzych-Fałta E., Wojas O., Samoliński B.K., Majsiak E., Białek S., Lishchuk-Yakymovych K. (2022). Gold standard diagnostic algorithm for the differential diagnosis of local allergic rhinitis. Postepy Dermatol. Alergol..

[12] Campo P., Eguiluz-Gracia I., Bogas G., Salas M., Plaza Serón C., Pérez N., Mayorga C., Torres M.J., Shamji M.H., Rondon C. (2019). Local allergic rhinitis: Implications for management. Clin. Exp. Allergy.

[13] Eguiluz-Gracia I., Pérez-Sánchez N., Bogas G., Campo P., Rondón C. (2019). How to Diagnose and Treat Local Allergic Rhinitis: A Challenge for Clinicians. J. Clin. Med..

[14] Krajewska-Wojtys A., Jarzab J., Zawadzińska K., Pyrkosz K., Bozek A. (2017). Local Allergic Rhinitis in Adult Patients with Chronic Nasal Symptoms. Int. Arch. Allergy Immunol..

[15] Bozek A., Scierski W., Ignasiak B., Jarzab J., Misiolek M. (2019). The prevalence and characteristics of local allergic rhinitis in Poland. Rhinology.

[16] Eguiluz-Gracia I., Fernandez-Santamaria R., Testera-Montes A., Ariza A., Campo P., Prieto A., Perez-Sanchez N., Salas M., Mayorga C., Torres M.J. (2020). Coexistence of nasal reactivity to allergens with and without IgE sensitization in patients with allergic rhinitis. Allergy.

[17] Papadopoulou A., Lambidi S., Lagousi T., Syrrou M., Giannoula F., Staikou E., Kostaridou S., Mermiri D.T. (2023). Nasal eosinophilia as a preliminary discriminative biomarker of non-allergic rhinitis in everyday clinical pediatric practice. Eur. Arch. Otorhinolaryngol..

[18] Augé J., Vent J., Agache I., Airaksinen L., Campo Mozo P., Chaker A., Cingi C., Durham S., Fokkens W., Gevaert P. (2018). EAACI Position paper on the standardization of nasal allergen challenges. Allergy.

[19] Cho S.H., Nanda A., Keswani A., Adinoff A., Baroody F.M., Bernstein J.A., Gherasim A., Han J.K., Koepke J.W., Ledford D.K. (2023). Rhinitis, Rhinosinusitis, and Ocular Allergy Committee of the AAAAI. Nasal allergen challenge (NAC): Practical aspects and applications from an EU/US perspective Work Group Report of the AAAAI Rhinitis, Rhinosinusitis and Ocular Allergy Committee. J. Allergy Clin. Immunol..

[20] Fauquert J.L., Alba-Linero C., Gherasim A., Testera-Montes A., Bentabol-Ramos G., Saenz de Santa Maria-Garcia R., Torres M.J., Eguiluz-Gracia I., Rondon C. (2023). Organ-specific allergen challenges in airway allergy: Current utilities and future directions. Allergy.

[21] Agache I., Bilò M., Braunstahl G.J., Delgado L., Demoly P., Eigenmann P., Gevaert P., Gomes E., Hellings P., Horak F. (2015). In vivo diagnosis of allergic diseases—Allergen provocation tests. Allergy.

[22] Eguiluz-Gracia I., Testera-Montes A., Salas M., Perez-Sanchez N., Ariza A., Bogas G., Bartra J., Torres M.J., Rondon C. (2021). Comparison of diagnostic accuracy of acoustic rhinometry and symptoms score for nasal allergen challenge monitoring. Allergy.

[23] Dramburg S., Hilger C., Santos A., de Las Vecillas L., Aalberse R.C., Acevedo N., Aglas L., Altmann F., Arruda K.L., Asero R. (2023). EAACI Molecular Allergology User’s Guide 2.0. Pediatr. Allergy Immunol..

[24] Bauer R.N., Xie Y., Beaudin S., Wiltshire L., Wattie J., Muñoz C., Alsaji N., Oliveria J.P., Ju X., MacLean J. (2023). Evaluation of the reproducibility of responses to nasal allergen challenge and effects of inhaled nasal corticosteroids. Clin. Exp. Allergy.

[25] Akerlund A., Andersson M., Leflein J., Lildholdt T., Mygind N. (2005). Clinical trial design, nasal allergen challenge models, and considerations of relevance to pediatrics, nasal polyposis, and different classes of medication. J. Allergy Clin. Immunol..

[26] Rondón C., Romero J.J., López S., Antúnez C., Martín-Casañez E., Torres M.J., Mayorga C., R-Pena R., Blanca M. (2007). Local IgE production and positive nasal provocation test in patients with persistent nonallergic rhinitis. J. Allergy Clin. Immunol..

[27] Rondón C., Doña I., López S., Campo P., Romero J.J., Torres M.J., Mayorga C., Blanca M. (2008). Seasonal idiopathic rhinitis with local inflammatory response and specific IgE in absence of systemic response. Allergy.

[28] López S., Rondón C., Torres M.J., Campo P., Canto G., Fernandez R., Garcia R., Martínez-Cañavate A., Blanca M. (2010). Immediate and dual response to nasal challenge with Dermatophagoides pteronyssinus in local allergic rhinitis. Clin. Exp. Allergy.

[29] Rondón C., Blanca-López N., Aranda A., Herrera R., Rodriguez-Bada J.L., Canto G., Mayorga C., Torres M.J., Campo P., Blanca M. (2011). Local allergic rhinitis: Allergen tolerance and immunologic changes after preseasonal immunotherapy with grass pollen. J. Allergy Clin. Immunol..

[30] Rondón C., Campo P., Galindo L., Blanca-López N., Cassinello M.S., Rodriguez-Bada J.L., Torres M.J., Blanca M. (2012). Prevalence and clinical relevance of local allergic rhinitis. Allergy.

[31] Chang G.U., Jang T.Y., Kim K.S., Choi H., Kim Y.H. (2014). Nonspecific hyper-reactivity and localized allergy: Cause of discrepancy between skin prick and nasal provocation test. Otolaryngol. Head. Neck Surg..

[32] Rondón C., Campo P., Zambonino M.A., Blanca-Lopez N., Torres M.J., Melendez L., Herrera R., Guéant-Rodriguez R.M., Guéant J.L., Canto G. (2014). Follow-up study in local allergic rhinitis shows a consistent entity not evolving to systemic allergic rhinitis. J. Allergy Clin. Immunol..

[33] Bozek A., Ignasiak B., Kasperska-Zajac A., Scierski W., Grzanka A., Jarzab J. (2015). Local allergic rhinitis in elderly patients. Ann. Allergy Asthma Immunol..

[34] Badran H.S., Hussein A., Salah M., Lotfi W.T. (2016). Identification and Prevalence of Allergic, Nonallergic, and Local Allergic Rhinitis Patients in Western Area, Saudi Arabia. Ann. Otol. Rhinol. Laryngol..

[35] ELBadawy N.E., El-Anwar M.W. (2016). Assessment of Nasal Immunoglobulin E Level in Atopic and Non-atopic Rhinitis Patients: A Tool for Diagnosis of Local Allergic Rhinitis. Egypt. J. Immunol..

[36] Blanca-Lopez N., Campo P., Salas M., García Rodríguez C., Palomares F., Blanca M., Canto G., Feo Brito F., Rondon C. (2016). Seasonal Local Allergic Rhinitis in Areas With High Concentrations of Grass Pollen. J. Investig. Allergol. Clin. Immunol..

[37] Rondón C., Blanca-López N., Campo P., Mayorga C., Jurado-Escobar R., Torres M.J., Canto G., Blanca M. (2018). Specific immunotherapy in local allergic rhinitis: A randomized, double-blind placebo-controlled trial with Phleum pratense subcutaneous allergen immunotherapy. Allergy.

[38] Rondon C., Campo P., Eguiluz-Gracia I., Plaza C., Bogas G., Galindo P., Mayorga C., Torres M.J. (2018). Local allergic rhinitis is an independent rhinitis phenotype: The results of a 10-year follow-up study. Allergy.

[39] Dykewicz M.S., Wallace D.V., Amrol D.J., Baroody F.M., Bernstein J.A., Craig T.J., Dinakar C., Ellis A.K., Finegold I., Golden D.B.K. (2020). Rhinitis 2020: A practice parameter update. J Allergy Clin Immunol..

[40] Jung C.G., Lee J.H., Ban G.Y., Park H.S., Shin Y.S. (2017). Prevalence and Clinical Characteristics of Local Allergic Rhinitis to House Dust Mites. Yonsei Med. J..

[41] Tao X.Y., Ng C.L., Chen D., Lin Z.B., Wu S.L., Liang M.J., Li C.W., Xu R. (2018). Clinical Characteristics and Allergen Sensitization Patterns of Patients with Local Allergic Rhinitis in Southern China. Int. Arch. Allergy Immunol..

[42] Shida M., Matsune S., Wakayama N., Ohashi R., Okubo K. (2020). Possibility of Local Allergic Rhinitis in Japan. Am. J. Rhinol. Allergy.

[43] Lipiec A., Sybilski A., Komorowski J., Furmańczyk K., Namysłowski A., Zieliński W., Raciborski F., Białoszewski A.Z., Samoliński B. (2020). Sensitisation to airborne allergens as a risk factor for allergic rhinitis and asthma in the Polish population. Postepy Dermatol Alergol..

[44] Tantilipikorn P., Siriboonkoom P., Sookrung N., Thianboonsong A., Suwanwech T., Pinkaew B., Asanasaen P. (2021). Prevalence of local allergic rhinitis to Dermatophagoides pteronyssinus in chronic rhinitis with negative skin prick test. Asian Pac. J. Allergy Immunol..

[45] Kim S.J., Moon J.W., Cho Y., Lee H.M. (2023). Clinical characteristics of local allergic rhinitis sensitized to house dust mites in Asia. Eur. Arch. Otorhinolaryngol..

[46] Maoz-Segal R., Machnes-Maayan D., Veksler-Offengenden I., Frizinsky S., Hajyahia S., Agmon-Levin N. (2019). Local Allergic Rhinitis: An Old Story but a New Entity.

[47] Sur D.K., Plesa M.L. (2015). Treatment of Allergic Rhinitis. Am. Fam. Physician.

[48] Fokkens W.J., Viskens A.-S., Backer V., Conti D., De Corso E., Gevaert P., Scadding G.K., Wagemann M., Bernal-Sprekelsen M., Chaker A. (2023). EPOS/EUFOREA update on indication and evaluation of Biologics in Chronic Rhinosinusitis with Nasal Polyps 2023. Rhinol. J..

[49] Hoang M.P., Samuthpongtorn J., Chitsuthipakorn W., Seresirikachorn K., Snidvongs K. (2022). Allergen-specific immunotherapy for local allergic rhinitis: A systematic review and meta-analysis. Rhinology.

[50] Campo P., Canonica G.W. (2024). Local Allergic Rhinitis. J. Allergy Clin. Immunol. Pract..

[51] Duman H., Bostanci I., Ozmen S., Dogru M. (2016). The Relevance of Nasal Provocation Testing in Children with Nonallergic Rhinitis. Int. Arch. Allergy Immunol..

[52] Krajewska-Wojtys A., Jarzab J., Gawlik R., Bozek A. (2016). Local allergic rhinitis to pollens is underdiagnosed in young patients. Am. J. Rhinol. Allergy.

[53] Zicari A.M., Occasi F., Di Fraia M., Mainiero F., Porzia A., Galandrini R., Giuffrida A., Bosco D., Bertin S., Duse M. (2016). Local allergic rhinitis in children: Novel diagnostic features and potential biomarkers. Am. J. Rhinol. Allergy.

[54] Colavita L., Catalano N., Sposito G., Loddo S., Galletti B., Salpietro C., Galletti F., Cuppari C. (2017). Local Allergic Rhinitis in Pediatric Patients: Is IgE Dosage in Nasal Lavage Fluid a Useful Diagnostic Method in Children?. Int. J. Mol. Cell Med..

[55] Tsilochristou O., Kyriakakou M., Manolaraki I., Lakoumentas J., Tiligada E., Maragkoudakis P., Douladiris N., Papadopoulos N.G. (2019). Detection of local allergic rhinitis in children with chronic, difficult-to-treat, non-allergic rhinitis using multiple nasal provocation tests. Pediatr. Allergy Immunol..

[56] Bożek A., Kozłowska R., Sybila P., Miodońska M., Mędrala A., Foks-Ciekalska A., Ignasiak B. (2023). The aspects of local allergic rhinitis in Polish children and adolescents. Postepy Dermatol. Alergol..

[57] Matsumoto F.Y., Tranquillini Gonçalves T.R., Solé D., Wandalsen G.F. (2024). Local allergic rhinitis in children: Identification and characterization in a specialty outpatient clinic. Eur. Ann. Allergy Clin. Immunol..

[58] Matsumoto F.Y., Gonçalves T.R.T., Solé D., Wandalsen G.F. (2022). Local allergic rhinitis in children: A systematic review. Allergol. Immunopathol..

[59] Costache A., Berghi O.N., Cergan R., Dumitru M., Neagos A., Popa L.G., Giurcaneanu C., Vrinceanu D. (2021). Respiratory allergies: Salicaceae sensitization (Review). Exp. Ther. Med..

[60] Vrinceanu D., Dumitru M., Patrascu O.M., Costache A., Papacocea T., Cergan R. (2021). Current diagnosis and treatment of rhinosinusal aspergilloma (Review). Exp. Ther. Med..

[61] Al-Ahmad M., Nurkic J., Bachert C., Pfaar O., Schunemann H.J., Czarlewski W., Bedbrook A., Bosquet J. (2021). ARIA 2019 Care Pathways for Allergic Rhinitis in the Kuwait Health Care System. Med. Princ. Pract..

[62] Arcangeli G., Traversini V., Tomasini E., Baldassarre A., Lecca L.I., Galea R.P., Mucci N. (2020). Allergic Anaphylactic Risk in Farming Activities: A Systematic Review. Int. J. Environ. Res. Public. Health.

